# Prevalence and Characteristics of Sleep Apnea in Patients With Heart Failure: A Single-Center Study

**DOI:** 10.7759/cureus.91358

**Published:** 2025-08-31

**Authors:** Bayyaram Rambhoopal Reddy, Jeetendra Kumar Patra, Manoranjan Pattnaik, Dipak Ranjan Das, Shanti Bhusan Das, Amit Kiran Rath, Sushant Kumar Nanda, Thejaswi N, Ahmed Rafad, Manoj Kumar Nayak

**Affiliations:** 1 Department of Pulmonary Medicine, Srirama Chandra Bhanja (SCB) Medical College and Hospital, Cuttack, IND; 2 Department of Cardiology, Srirama Chandra Bhanja (SCB) Medical College and Hospital, Cuttack, IND; 3 Department of Radiology, Srirama Chandra Bhanja (SCB) Medical College and Hospital, Cuttack, IND

**Keywords:** apnea hypopnea index, central sleep apnea, heart failure, left ventricular ejection fraction, obstructive sleep apnea

## Abstract

Introduction

Heart failure is a common outcome of chronic cardiovascular diseases and carries a poor prognosis. Sleep apnea is an important comorbidity in heart failure patients, increasing the risk of mortality. Obstructive sleep apnea (OSA) is more commonly associated with heart failure with preserved ejection fraction, while central sleep apnea (CSA) prevalence increases with worsening New York Heart Association (NYHA) functional class and decreasing ejection fraction. The primary endpoint of the study is the prevalence of sleep apnea in heart failure patients. Secondary endpoints were the characteristics of sleep apnea in heart failure, the association between sleep apnea and heart failure severity, and the relationship between sleep apnea and symptoms.

Methods

This single-center, cross-sectional study was conducted in the Department of Pulmonary Medicine and Cardiology, SCB Medical College and Hospital, Cuttack, Odisha, from March 2023 to July 2024. Fifty consenting adult, stable heart failure patients were included. All patients were on appropriate pharmacological therapy, with no acute exacerbation or medication change for over 1 month.

Exclusion criteria

Patients with NYHA class IV, resting hypoxia, history of myocardial infarction, unstable angina, cardiac surgery within the past 3 months, pregnancy, obstructive lung disease, or hypothyroidism were excluded from the study. Heart failure was diagnosed based on clinical signs and symptoms and the assessment of left ventricular function by 2D echocardiography. Overnight polysomnography was performed to diagnose and assess sleep apnea.

Results

Our study included 50 heart failure patients with a mean age of 54.84 years (±15.12) and a mean BMI of 27.36 kg/m² (±6.55). The cohort comprised 23 patients with heart failure with preserved ejection fraction, 8 with mildly reduced ejection fraction, and 19 with reduced ejection fraction. All patients experienced dyspnea, while 94% reported fatigue. Common comorbidities included hypertension (72%) and diabetes (44%). The mean apnea-hypopnea index (AHI) was 28.39 (±26.33), with an apnea index of 19.71 (±20.80) and hypopnea index of 8.48 (±13.87). Sleep apnea affected 86% of patients, with 68% having OSA and 18% having CSA. Female patients had a higher prevalence of sleep apnea (90.5%) than males (82.75%). Sleep apnea was common across all heart failure categories, with OSA predominant in each. Positive correlations were found between AHI and total sleep time below 90% oxygen saturation (TST90), while negative correlations were found between AHI and minimum SpO₂ during sleep. However, multivariate regression analysis revealed that BMI, left ventricular ejection fraction, age, and male gender were not independent risk factors for sleep apnea severity.

Conclusion

This study demonstrates a high prevalence of sleep apnea among stable heart failure patients, with OSA being the most common type. Sleep apnea was common across all heart failure categories, with a higher prevalence in female patients. The findings highlight the significant association between heart failure and sleep apnea, emphasizing the need for increased awareness and screening. The study’s results have important implications for the diagnosis and treatment of sleep apnea in heart failure patients.

## Introduction

Heart failure (HF) is a common outcome of various chronic cardiovascular diseases, including hypertension, coronary artery disease, valvular heart disease, and cardiomyopathy. The prevalence of HF increases significantly with age, affecting 1-2% of adults aged 40-59 years and up to 10% of those over 70 years in India [1]. Despite advances in treatment, HF carries a poor prognosis, with a 5-year survival rate of 50% and a 1-year mortality rate of 40% in stage D HF [2]. HF can be further divided into three types based on ejection fraction: HF with preserved ejection fraction (HFpEF), HF with mildly reduced ejection fraction (HFmrEF), and HF with reduced ejection fraction (HFrEF).

Sleep apnea is a common comorbidity in HF patients, with a prevalence of 50-75% [3]. Two types of sleep apnea are seen in HF: obstructive sleep apnea (OSA) and central sleep apnea (CSA). OSA is more commonly associated with HFpEF, while CSA prevalence increases with worsening New York Heart Association (NYHA) functional class and decreasing ejection fraction [4,5]. Sleep apnea is an independent risk factor for increased mortality in HF, and its underdiagnosis is attributed to overlapping symptoms between sleep apnea and HF [6].

An intricate pathophysiological interplay exists between sleep apnea and HF, characterized by multifactorial mechanisms. During sleep, fluid redistribution precipitates pharyngeal edema, while augmented circulatory time and pulmonary congestion culminate in CSA [5,7]. Moreover, recurrent intrathoracic pressure oscillations trigger sympathetic nervous system activation, eliciting inflammatory responses and endothelial damage [8-10]. These converging factors collectively contribute to metabolic derangements, cardiac dysfunction, and heightened susceptibility to sudden cardiac mortality. CSA in HF is associated with worse prognosis, as increased sympathetic activity leads to atrial fibrillation (AF) and sudden cardiac death [11]. 

Sleep apnea worsens HF outcomes but is often underdiagnosed due to overlapping symptoms, making its detection important for better management. Data on the prevalence and characteristics of sleep apnea in different types of HF, especially in Indian patients, are limited. The primary objective of this study was to determine the prevalence of sleep apnea in HF patients. Secondary objectives were to describe the characteristics of sleep apnea in HF, assess its association with HF severity, and evaluate its relationship with symptoms.

This study aims to investigate the prevalence of sleep apnea in HF in Eastern India, addressing the scarcity of data in this region.

## Materials and methods

This study was a single-centre, cross-sectional study undertaken in the Department of Pulmonary Medicine and Cardiology, SCB Medical College and Hospital, Cuttack, Odisha, from March 2023 to July 2024, after approval by the Institutional Ethical Committee (IEC). The sample size was calculated using the prevalence study formula. Most previous studies showed a prevalence rate ranging from 50% to 80%. A single-centre study published by Kishan S et al. reported the prevalence of sleep apnea in heart failure as 82%. We took prevalence (p) as 82%, level of confidence (z) as 1.96, and precision (d) as 10% for sample size calculation. The calculated sample size was 56; we took a sample size of 50 for technical reasons.

All participants were recruited after providing written informed consent. Fifty consecutive consenting adult, stable HF patients were included, all receiving appropriate pharmacological therapy with no acute exacerbation or medication/dose change for over 1 month. This study was performed in patients referred to the Pulmonary Medicine Sleep Clinic by the Cardiology Department. Heart failure was diagnosed by a cardiologist on the basis of clinical symptoms, signs, and 2D echocardiography findings. HF was subdivided into three categories based on 2D echocardiography findings. The exclusion criteria were NYHA class IV, resting hypoxia, history of myocardial infarction, unstable angina, cardiac surgery within 3 months, pregnancy, obstructive lung disease, hypothyroidism, and all other risk factors for sleep apnea. Demographic and anthropometric data, including age, sex, BMI, neck circumference, and blood pressure, were recorded. Overnight polysomnography (PSG) was performed using video polysomnography (SU1000-64LW, Sienna 64, EMS Biomedical) as per the 2023 American Academy of Sleep Medicine (AASM) guidelines, by a certified polysomnographic technologist. Manual scoring was used to report respiratory and sleep events. The PSG analyst was blinded to the HF status of the patient (single-blinded study). The AHI was used to diagnose sleep apnea and assess OSA severity, with participants categorized into non-OSA (AHI ≤5), mild (5 < AHI ≤15), moderate (15 < AHI ≤30), and severe (AHI >30) OSA groups. CSA was differentiated from OSA by the absence of respiratory effort (thoraco-abdominal movements).

Statistical analysis was performed using SPSS version 20 software, applying chi-square tests, Student’s t-tests, ANOVA, Pearson’s and Spearman’s correlation, and univariate and multivariable logistic regression analysis.

## Results

Our study cohort of 50 HF patients comprised 23 patients with HFpEF, eight patients with HFmrEF, and 19 patients with HFrEF. All patients experienced dyspnea, while 94% (47) reported fatigue. Common sleep-related symptoms included arousals in 60% (30), snoring in 50% (25), and nocturia in 28% (14). Excessive daytime sleepiness was reported by 48% of patients (24/50). Hypertension 72% (36) and diabetes mellitus 44% (22) were the most common comorbidities. Underlying cardiac conditions included dilated cardiomyopathy (DCM) 30% (15) and ischemic heart disease (IHD) 18% (9), both more commonly associated with HFrEF. The mean age of the study patients was 54.84 ± 15.12 years, with a significant difference between males and females (50.89 ± 13.26 vs. 60.28 ± 16.14, p = 0.02). The mean BMI of the study subjects was 27.36 ± 6.55 kg/m². Male HF patients had higher mean BMI, neck circumference, and waist circumference compared to female patients. Significant differences in BMI and waist circumference were found among HFpEF, HFmrEF, and HFrEF subgroups (p = 0.01 and p = 0.04) (Table 1).

**Table 1:** Clinical and demographic details of study patients. HFpEF: Heart Failure with Preserved Ejection Fraction; HFmrEF: Heart Failure with Mildly Reduced Ejection Fraction; HFrEF: Heart Failure with Reduced Ejection Fraction; BMI: Body Mass Index; NYHA: New York Heart Association.

Parameter	HFpEF (N=23)	HFmrEF (N=8)	HFrEF (N=19)	Total (N=50)	Chi-square	ANOVA	p-value
Males, n (%)	10 (43.5)	5 (62.5)	14 (73.7)	29 (58.0)	3.99	NA	0.14
Females, n (%)	13 (56.5)	3 (37.5)	5 (26.3)	21 (42.0)	-	-	-
Age, mean ± SD	54.56 ± 15.86	49.00 ± 10.02	57.63 ± 16.49	54.84 ± 15.12	NA	0.92	0.4
BMI, mean ± SD	29.92 ± 6.18	27.26 ± 6.99	24.31 ± 5.73	27.36 ± 6.55	NA	4.34	0.01*
Waist circumference, mean ± SD	94.08 ± 14.17	87.00 ± 14.30	83.52 ± 12.43	88.94 ± 14.16	NA	3.25	0.04*
Neck circumference, mean ± SD	39.19 ± 3.25	38.00 ± 3.58	37.07 ± 3.50	38.19 ± 3.47	NA	2.02	0.14
Fatigue, n (%)	21 (91.3)	7 (87.5)	19 (100)	47 (94.0)	2.11	NA	0.34
Snoring, n (%)	14 (61.0)	5 (62.5)	6 (31.6)	25 (50.0)	3.29	NA	0.19
Nocturia, n (%)	6 (26.1)	2 (25.0)	6 (31.6)	14 (28.0)	0.2	NA	0.9
Witnessed apneas, n (%)	7 (30.4)	2 (25.0)	3 (15.8)	12 (24.0)	1.23	NA	0.54
Choking spells, n (%)	7 (30.4)	2 (25.0)	3 (15.8)	12 (24.0)	1.23	NA	0.54
Arousals, n (%)	16 (69.6)	7 (87.5)	7 (36.8)	30 (60.0)	8	NA	0.02*
NYHA I, n (%)	5 (22)	1 (12.5)	3 (15.8)	9 (18.0)	0.66	NA	0.96
NYHA II, n (%)	12 (52)	4 (50.0)	10 (52.6)	26 (52.0)	-	-	-
NYHA III, n (%)	6 (26.1)	3 (37.5)	6 (31.6)	15 (30.0)	-	-	-
Alcohol consumption, n (%)	2 (8.7)	0	3 (15.8)	5 (10.0)	1.64	NA	0.44
Smoking, n (%)	3 (13.0)	0	4 (21.1)	7 (14.0)	2.1	NA	0.35
Hypertension, n (%)	17 (73.9)	7 (87.5)	12 (63.2)	36 (72.0)	1.73	NA	0.42
Diabetes mellitus, n (%)	8 (34.8)	2 (25.0)	12 (63.2)	22 (44.0)	4.8	NA	0.09
Dilated cardiomyopathy, n (%)	0	2 (25.0)	13 (68.4)	15 (30.0)	23.32	NA	<0.001*
Hyperlipidemia, n (%)	6 (26.1)	4 (50.0)	2 (10.5)	12 (24.0)	4.91	NA	0.08
Ischemic heart disease, n (%)	1 (4.3)	2 (25.0)	6 (31.6)	9 (18.0)	5.54	NA	0.06
Chronic kidney disease, n (%)	0	0	2 (10.5)	2 (4.0)	3.4	NA	0.18
Cerebrovascular disease, n (%)	1 (4.3)	1 (12.5)	0	2 (4.0)	2.3	NA	0.32
Atrial fibrillation, n (%)	0	0	1 (5.3)	1 (2.0)	1.67	NA	0.44

Diuretics 72% (36), angiotensin II receptor blockers (ARBs) 68% (34), beta-blockers 50% (25), and oral hypoglycemic agents (OHA) 50% (25) were the most commonly used medications, with higher usage in HFrEF patients (Table 2).

**Table 2:** Medication use among heart failure patients. ACEI: Angiotensin-Converting Enzyme Inhibitor; ARB: Angiotensin Receptor Blocker; CCB: Calcium Channel Blocker; OHA: Oral Hypoglycemic Agent; SGLT2i: Sodium-Glucose Cotransporter-2 Inhibitor.

Medication	HFpEF (N=23)	HFmrEF (N=8)	HFrEF (N=19)	Total (N=50)	Chi-square	ANOVA	p-value
Diuretics, n (%)	14 (60.9)	6 (75.0)	16 (84.2)	36 (72.0)	2.86	NA	0.24
ACEI, n (%)	2 (8.7)	3 (37.5)	9 (47.4)	14 (28.0)	8.15	NA	0.02*
ARB, n (%)	16 (69.6)	5 (62.5)	13 (68.4)	34 (68.0)	0.14	NA	0.93
Antiplatelets, n (%)	2 (8.7)	3 (37.5)	6 (31.6)	11 (22.0)	4.48	NA	0.1
Statins, n (%)	4 (17.4)	5 (62.5)	7 (36.8)	16 (32.0)	5.93	NA	0.05
Anticoagulants, n (%)	0	0	2 (10.5)	2 (4.0)	3.4	NA	0.18

Polysomnography demonstrated a mean AHI of 28.39 ± 26.33 events/hour with a median of 16.44, indicating a right-skewed distribution due to a subset of patients with markedly elevated values. The mean apnea index was 19.71 ± 20.80 (median 12.54), while the mean hypopnea index was 8.48 ± 13.87 (median 2.65), suggesting that apneic events contributed more substantially to the overall AHI. The mean minimum SpO₂ recorded during sleep was 77.20 ± 15.08%, reflecting significant nocturnal desaturation in the study population. Sleep apnea affected 86% of patients (43/50), with 68% having OSA (34/50) and 18% having CSA (9/50).

Female HF patients had a higher prevalence of sleep apnea (90.5%, 19/21) than males (82.75%, 24/29, p = 0.12), with OSA more common in females (80.95%, 17/21) and CSA more common in males (24.13%, 7/29, p = 0.14). Sleep apnea prevalence was high across HF categories: HFpEF (82.6%, 19/23), HFmrEF (87.5%, 7/8), and HFrEF (89.5%, 17/19) (p = 0.59), with OSA predominant in all (p = 0.19). Additionally, severe OSA patients had lower sleep efficiency; arrhythmias were more frequent in CSA and severe OSA patients; CSA patients were older; severe OSA patients had higher BMI; and CSA patients had lower neck and waist circumference and BMI compared to OSA patients.

Common sleep apnea symptoms included nighttime arousals 60% (30), snoring 50% (25), nocturia 28% (14), witnessed apneas 24% (12), and choking spells 24% (12). No significant differences were found in clinical presentation, pedal edema, vital signs, oxygen saturation, or comorbidities between HF patients with and without sleep apnea. The most commonly used medications were OHA 74% (37), diuretics 72% (36), ARBs 68% (34), and beta-blockers 50% (25) (Table 3).

**Table 3:** Demographic and clinical characteristics among patients with no sleep apnea, OSA, and CSA with heart failure. NSA: No Sleep Apnea; OSA: Obstructive Sleep Apnea; CSA: Central Sleep Apnea; WC: Waist Circumference; NC: Neck Circumference; LVEF: Left Ventricular Ejection Fraction; HFpEF: Heart Failure with Preserved Ejection Fraction; HFmrEF: Heart Failure with Mildly Reduced Ejection Fraction; HFrEF: Heart Failure with Reduced Ejection Fraction; HTN: Hypertension; PHTN: Pulmonary Hypertension; DM: Diabetes Mellitus; DCM: Dilated Cardiomyopathy; IHD: Ischemic Heart Disease; NYHA: New York Heart Association; ACEI: Angiotensin-Converting Enzyme Inhibitor; ARB: Angiotensin Receptor Blocker; CCB: Calcium Channel Blocker; OHA: Oral Hypoglycemic Agent; SGLT2i: Sodium-Glucose Cotransporter-2 Inhibitor; NA: Not Applicable. p < 0.05 was considered statistically significant.

Parameter	NSA (n=7)	OSA (n=34)	CSA (n=9)	Total (N=50)	Chi-square	ANOVA	p-value
Age, Mean ± SD	57.57 ± 15.65	53.97 ± 15.86	56.00 ± 12.95	54.84 ± 15.12	NA	0.19	0.83
Males n (%)	5 (71.4)	17 (50.0)	7 (77.8)	29 (58.0)	2.85	NA	0.24
Females n (%)	2 (28.6)	17 (50.0)	2 (22.2)	21 (42.0)	-	-	-
BMI, Mean ± SD	25.17 ± 5.12	28.51 ± 6.91	24.75 ± 5.38	27.36 ± 6.55	NA	1.67	0.2
WC, Mean ± SD	83.29 ± 6.29	92.02 ± 12.66	81.67 ± 8.75	88.94 ± 14.16	NA	2.73	0.07
NC, Mean ± SD	37.43 ± 1.81	38.70 ± 2.63	36.89 ± 3.44	38.19 ± 3.47	NA	1.18	0.32
LVEF, Mean ± SD	50.57 ± 11.60	48.82 ± 12.55	42.44 ± 11.01	47.92 ± 13.88	NA	0.89	0.42
HFpEF n (%)	4 (57.1)	17 (50.0)	2 (22.2)	23 (46.0)	3.76	NA	0.44
HFmrEF n (%)	1 (14.3)	4 (11.8)	3 (33.3)	8 (16.0)	-	-	-
HFrEF n (%)	2 (28.6)	13 (38.2)	4 (44.4)	19 (38.0)	-	-	-
Dilated LA n (%)	2 (28.6)	10 (29.4)	6 (66.7)	18 (36.0)	4.49	NA	0.1
PHTN n (%)	2 (28.6)	8 (23.5)	2 (22.2)	12 (24.0)	0.09	NA	0.95
Fatigue n (%)	7 (100)	31 (91.2)	9 (100)	47 (94.0)	1.83	NA	0.47
Arousals n (%)	3 (42.9)	22 (64.7)	5 (55.6)	30 (60.0)	1.24	NA	0.53
Snoring n (%)	2 (28.6)	20 (58.8)	3 (33.3)	25 (50.0)	3.34	NA	0.18
Witnessed apneas n (%)	0	10 (29.4)	2 (22.2)	12 (24.0)	3.73	NA	0.25
Choking spells n (%)	0	10 (29.4)	2 (22.2)	12 (24.0)	3.73	NA	0.25
Nocturia n (%)	0	11 (32.3)	3 (33.3)	14 (28.0)	3.17	NA	0.2
Mood disturbances n (%)	0	3 (8.8)	0	3 (6.0)	1.5	NA	0.47
NYHA I n (%)	1 (14.3)	5 (14.7)	3 (33.3)	9 (18.0)	3.22	NA	0.52
NYHA II n (%)	5 (71.4)	18 (52.9)	3 (33.3)	26 (52.0)	-	-	-
NYHA III n (%)	1 (14.3)	11 (32.3)	3 (33.3)	15 (30.0)	-	-	-
Pedal edema n (%)	3 (42.9)	17 (50.0)	6 (66.7)	26 (52.0)	1.06	NA	0.59
HTN n (%)	6 (85.7)	24 (70.6)	6 (66.7)	36 (72.0)	0.81	NA	0.67
DM n (%)	1 (14.3)	16 (47.1)	5 (55.6)	22 (44.0)	3.12	NA	0.21
Dyslipidemia n (%)	0	10 (29.4)	2 (22.2)	12 (24.0)	2.76	NA	0.25
DCM n (%)	0	11 (32.3)	4 (44.4)	15 (30.0)	4	NA	0.14
IHD n (%)	3 (42.9)	5 (14.7)	1 (11.1)	9 (18.0)	3.46	NA	0.18
Diuretics n (%)	3 (42.9)	25 (73.5)	8 (88.9)	36 (72.0)	4.26	NA	0.12
ACEI n (%)	2 (28.6)	10 (29.4)	2 (22.2)	14 (28.0)	0.18	NA	0.91
ARB n (%)	3 (42.9)	24 (70.6)	7 (77.8)	34 (68.0)	2.53	NA	0.28
Statins n (%)	3 (42.9)	12 (35.3)	1 (11.1)	16 (32.0)	2.35	NA	0.31
Antiplatelets n (%)	4 (57.1)	6 (17.6)	1 (11.1)	11 (22.0)	6.03	NA	0.04*
Beta-blockers n (%)	4 (57.1)	18 (52.9)	3 (33.3)	25 (50.0)	1.26	NA	0.53
CCB n (%)	1 (14.3)	6 (17.6)	3 (33.3)	10 (20.0)	1.26	NA	0.53
OHA n (%)	4 (57.1)	10 (29.4)	3 (33.3)	17 (34.0)	1.99	NA	0.37
Insulin n (%)	0	4 (11.8)	3 (33.3)	7 (14.0)	4.11	NA	0.13
SGLT2i n (%)	2 (28.6)	13 (38.2)	5 (55.6)	20 (40.0)	2.11	NA	0.35

Multivariate regression analysis revealed that BMI, LVEF, age, and male gender were not independent risk factors for sleep apnea severity (Table 4).

**Table 4:** Multivariate logistic regression analysis of AHI, adjusted for BMI, LVEF, age, and gender. LVEF: Left Ventricular Ejection Fraction.

Parameters	Odds Ratio	95% CI Lower	95% CI Upper	p-value
BMI	1.095	0.977	1.227	0.118
Age	1.012	0.971	1.055	0.568
LVEF	0.962	0.911	1.015	0.158
Sex (male)	0.927	0.241	3.57	0.912

Univariate analysis showed that female age was also not an independent risk factor (Table 5).

**Table 5:** Univariate analysis between female age and AHI. AHI: Apnea-Hypopnea Index. p < 0.05 was considered statistically significant.

	Coefficients	Standard Error	t Stat	p-value	Lower 95%	Upper 95%
Intercept	56.59828	5.024802	11.26378	7.49E-10	46.08125	67.11531
AHI	0.121939	0.118669	1.027552	0.317065	-0.12644	0.370316

Additionally, LVEF did not correlate with AHI, oxygen desaturation index (ODI), total sleep time with oxygen saturation below 90% (TST90), mean SpO₂, or minimum SpO₂ during sleep (Table 6).

**Table 6:** Correlation of LVEF with PSG parameters . LVEF: Left Ventricular Ejection Fraction; PSG: Polysomnography; AHI: Apnea–Hypopnea Index; ODI: Oxygen Desaturation Index; TST90: Total Sleep Time with oxygen saturation below 90%; AROINX: Arousal Index. p < 0.05 was considered statistically significant.

Correlation	AHI	ODI	Mean SpO₂	Minimum SpO₂	TST90	AROINX
LVEF (r)	0.02	0.003	-0.150	-0.172	0.003	0.082
p	0.891	0.981	0.3	0.234	0.983	0.573

**Figure 1:**
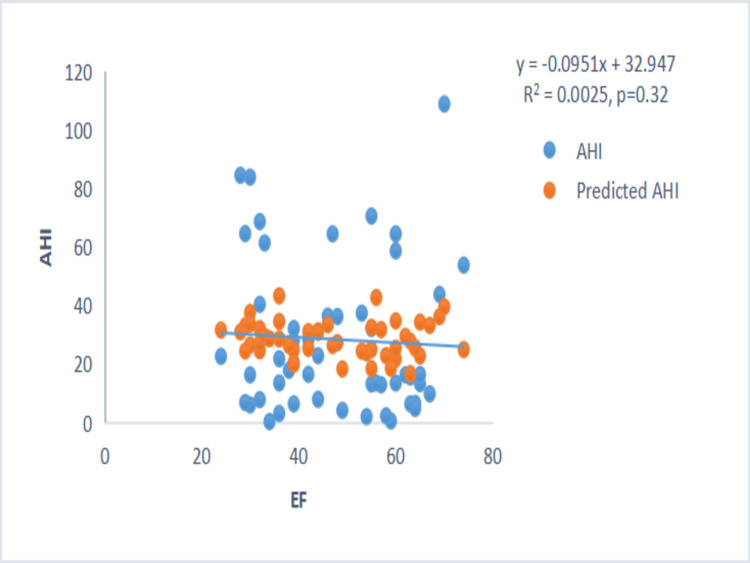
Scatter plot between AHI and LVEF. Scatter plot showing no correlation between AHI and LVEF. AHI: Apnea-Hypopnea Index; LVEF: Left Ventricular Ejection Fraction.

**Figure 2:**
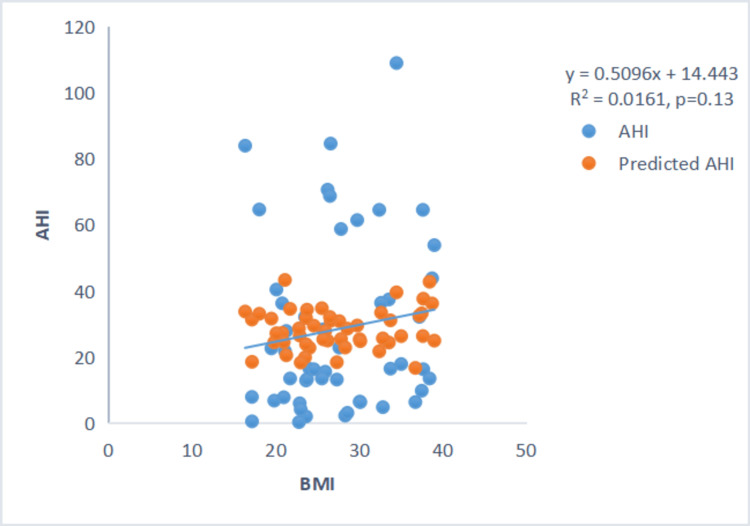
Scatter plot between AHI and BMI. Scatter plot showing no correlation between AHI and BMI. AHI: Apnea-Hypopnea Index.

There was no correlation between the severity of sleep apnea (AHI) and LVEF or BMI (Figures 1-2).

**Figure 3:**
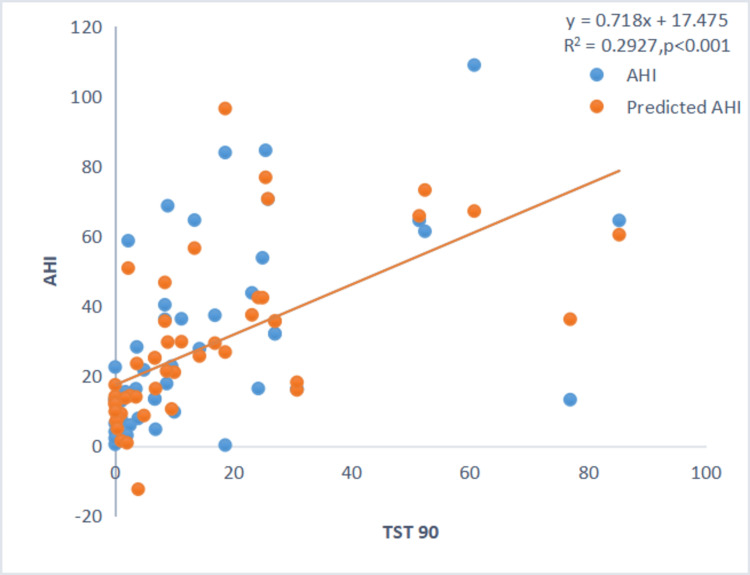
Scatter plot between AHI and TST90. Scatter plot showing a positive correlation between AHI and TST90. AHI: Apnea-Hypopnea Index; TST90: Total Sleep Time with oxygen saturation below 90%.

**Figure 4:**
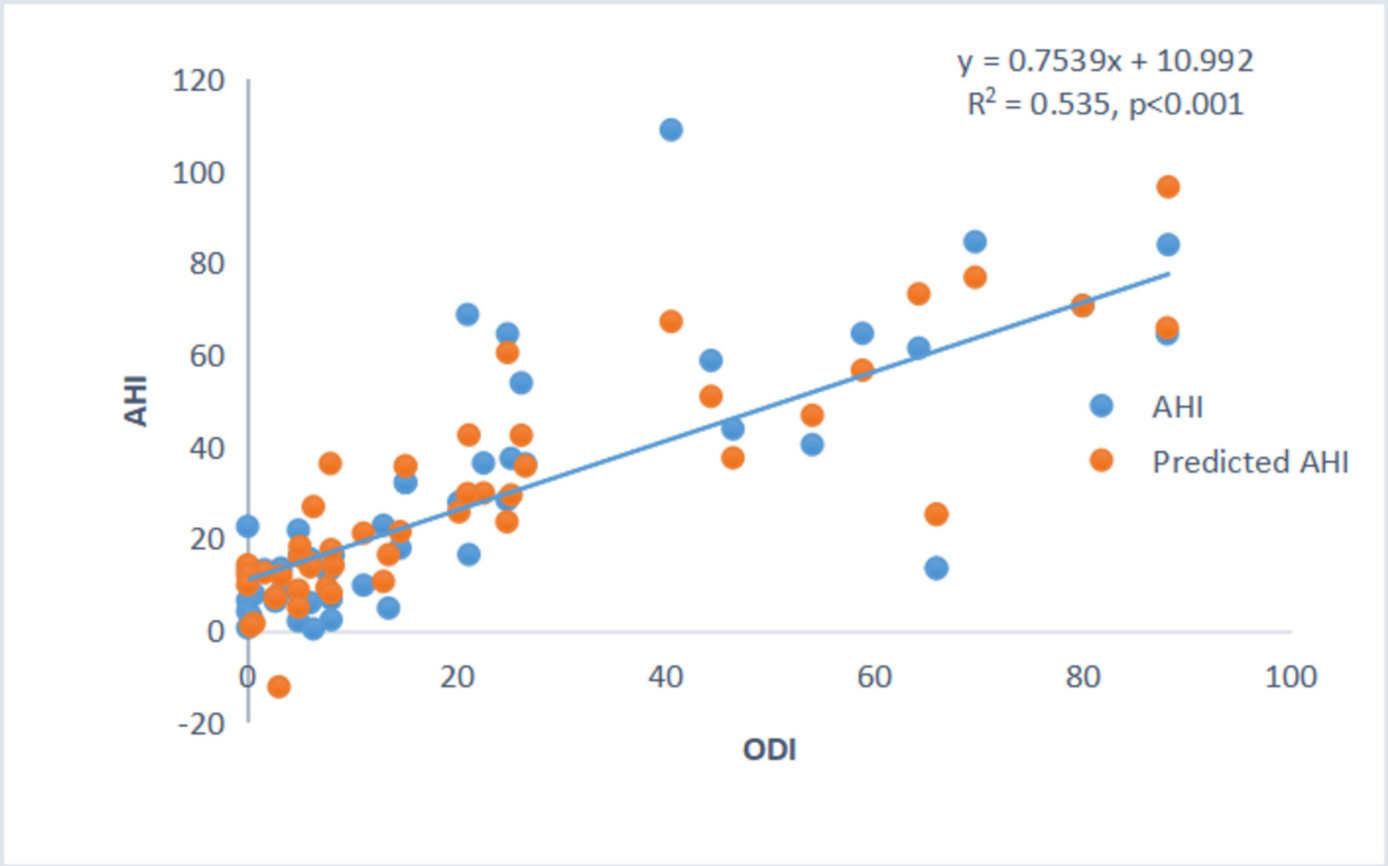
Scatter plot between AHI and ODI. Scatter plot showing a positive correlation between AHI and ODI. AHI: Apnea-Hypopnea Index; ODI: Oxygen Desaturation Index.

However, the severity of sleep apnea (AHI) showed a positive correlation with TST90 and ODI, and a negative correlation with minimum SpO₂ during sleep (Figures 3-4).

## Discussion

We enrolled stable HF patients regardless of their sleep apnea symptom status, similar to studies by Kishan S et al., Schulz R et al., and Arzt M et al. [1,12,13]. The mean age was 54.84 years, with males (58%) being younger (50.89 years) than females (60.28 years). Males also had a higher mean BMI (28.21 kg/m²) than females (25.80 kg/m²). The baseline characteristics of our study population are comparable to those in Yumino et al.’s study [14].

Our study uncovered a strikingly high rate of sleep apnea among HF patients, with 86% (43/50) affected. OSA was the most common type, impacting 68% (34/50) of patients, while CSA affected 18% (9/50). This prevalence far exceeds that of the general population [12], highlighting a significant association between HF and sleep apnea. Studies by Kishan S et al. (82%), Oldenburg O et al. (71%), Schulz R et al. (76%), and Isakson SR et al. (85%) reported similar prevalence rates of sleep apnea in chronic HF patients [1,6,12,15]. Our study’s finding of 50% moderate-to-severe sleep apnea is comparable to Sin DD et al.’s result of 61% (AHI cutoff 15) in 450 stable HF patients [16]. These studies consistently demonstrate a high prevalence of sleep apnea in HF patients, with OSA being the predominant type. Bitter et al. reported a 69.3% prevalence of sleep apnea in HFpEF patients, with OSA in 39.8% [4]. Yumino et al. found a 47% prevalence of moderate-to-severe sleep apnea in HF patients [14]. Singh P et al. reported 72% prevalence of sleep apnea in HF patients, although different patient characteristics may have contributed to the variation [17]. Collectively, these studies highlight the high burden of sleep apnea in HF patients and emphasize the need for increased awareness and screening.

Research by Schulz R et al., Herrscher et al., and Kalaydzhiev P et al. reported OSA rates in HF patients ranging from 43% to 82% [12,18,19]. Our study found a similarly high OSA rate of 68%, with CSA at 18%. However, other studies have reported varying prevalence patterns, with some identifying a more balanced distribution of OSA and CSA or a higher prevalence of CSA. Oldenburg O et al. reported OSA and CSA rates of 32% and 34%, respectively, while Singh P et al. and Vazir A et al. found higher CSA rates of 38% and 60.5% compared to OSA rates of 15% and 39.5% [6,17,20]. The disparity in results may be attributed to differences in patient characteristics, such as age, LVEF, and AF, which were more prevalent in studies with higher CSA rates. In contrast, our study found a higher prevalence of OSA, potentially due to higher BMI and pedal edema in OSA patients.

Our study also revealed a high prevalence of sleep apnea across all HF subtypes, with rates of 82.6% in HFpEF, 87.5% in HFmrEF, and 89.5% in HFrEF. OSA was the most common subtype, affecting 73.9% of HFpEF, 50% of HFmrEF, and 68.5% of HFrEF patients, whereas CSA was more prevalent in HFmrEF (37.5%) and HFrEF (21%) compared to HFpEF (8.7%). Similar studies by Wang T et al. and Borrelli C et al. reported varying prevalence rates, likely due to differences in baseline characteristics [21,22]. A systematic review by Piccirillo F et al. reported a prevalence range of 50-75% for sleep apnea, with CSA being the predominant type in 45-55% of cases [3]. Our findings corroborate the association between decreasing LVEF, worsening cardiac function, and increased CSA prevalence. However, prior research on HF patients with reduced ejection fraction has yielded inconsistent results, highlighting the complexity of this relationship.

Kazimierczak A et al. reported an inverse relationship between LVEF and CSA prevalence, finding that lower LVEF was associated with increased CSA prevalence [5]. However, other studies, including ours and that of Arzt M et al. [13], found no significant correlation between LVEF and sleep-disordered breathing severity. In contrast, Schulz R et al. and Javaheri S et al. observed that lower LVEF increased the risk of sleep apnea, particularly CSA [12,23]. The discrepancies in findings may be attributed to differences in study populations, with some studies focusing exclusively on HFrEF.

Lanfranchi PA et al. observed a significant association between severe CSA and increased cardiac arrhythmias in HF patients [11]. Similarly, our study found a higher frequency of cardiac arrhythmias in CSA patients, with 66.7% showing increased left atrial size. In contrast, Arzt M et al. reported a different distribution of sleep apnea types in HFrEF, with 31% having CSA, 29% OSA, and 40% mixed sleep apnea, and periodic breathing in 41% of cases [24]. Our study, however, found OSA to be the prevailing type in HFmrEF and HFrEF, which may be influenced by beta-blocker therapy, consistent with earlier research by Schulz R et al. and Paulino A et al. [12,25]. Notably, our study did not identify any cases of mixed sleep apnea but did find a high prevalence of pedal edema in CSA patients, suggesting that factors such as circulatory delay and rostral fluid shift may contribute to CSA development.

In our study, female HF patients had a higher overall prevalence of sleep apnea (90.5% vs. 83%) but a lower prevalence of CSA (9.5% vs. 24.5%) compared to males. The lower prevalence of CSA in females may be influenced by hormonal factors. Although our multivariate analysis did not find a correlation between sleep apnea severity and age or male sex, previous studies suggest that older age and male gender are associated with increased prevalence of sleep apnea [12,13,25]. Interestingly, in our study, age was a significant predictor of OSA in females, despite no correlation between age and AHI in univariate analysis.

BMI was higher in patients with OSA, consistent with Sin DD et al., who found that BMI is a risk factor specifically for OSA in congestive HF patients [16]. However, multivariate regression analysis did not reveal a correlation between BMI and AHI. This finding contrasts with Schulz R et al. [12] but supports Arzt M et al.’s observation that HF patients tend to have lower BMIs for any given AHI value [26]. The development of OSA in HF patients may therefore be influenced by factors such as nocturnal rostral fluid shift, rather than BMI alone. We also investigated the association between waist and neck circumference and sleep apnea. While both were generally higher in patients with OSA, subgroup analysis showed a significant difference only in female patients with severe OSA. This finding differs from studies in non-HF populations [27,28], suggesting that HF itself may alter the relationship between body habitus and OSA.

All patients in our study had exertional dyspnea due to HF, and the majority (94%) reported fatigue, a symptom common to both HF and sleep apnea. Other symptoms, including nocturnal arousals, snoring, nocturia, and witnessed apneas, were also prevalent, but their frequency did not differ significantly between patients with and without sleep apnea. In HF patients, arousals can be triggered by paroxysmal nocturnal dyspnea or obstructive apneas, while nocturia may be caused by increased renal hemodynamics or atrial natriuretic peptide release due to apneas. Previous studies have identified specific symptoms associated with sleep apnea in HF patients, such as witnessed apneas and fatigue as predictors of CSA, and snoring as an indicator of OSA [13,29]. Our study also found that mood disturbances were more prevalent in patients with sleep apnea, consistent with Parati G et al. [30]. However, we did not find a correlation between sleep apnea severity and worsening NYHA functional class, which aligns with the findings of Schulz R et al. [12] but differs from those of Arzt M et al. [13].

Limitations

This study has several limitations. First, the sample size was small. Second, it was conducted at a single tertiary care center, which may introduce selection bias, such as referral bias. Third, the cross-sectional design limits the ability to establish causal relationships between OSA and HF. Finally, the study did not include follow-up or outcome data and therefore could not assess the impact of OSA on HF prognosis or outcomes, such as readmissions or mortality.

## Conclusions

This study demonstrates a very high prevalence of sleep apnea (86%) among stable HF patients, with OSA being the predominant type across all HF phenotypes. Sleep apnea was common irrespective of ejection fraction and was more frequent in females, with OSA associated with higher BMI and CSA linked to older age, arrhythmias, and atrial enlargement. The severity of sleep apnea correlated with nocturnal hypoxemia but not with LVEF, BMI, or gender. Symptomatically, almost all patients experienced dyspnea and fatigue, while sleep-related complaints such as nocturnal arousals, snoring, nocturia, witnessed apneas, and choking spells were also frequent, though not significantly different between patients with and without sleep apnea. These findings highlight the under-recognized burden of sleep apnea in HF and underscore the need for routine screening and integrated management to improve patient outcomes.
